# Biomedical applications of copper-free click chemistry: *in vitro*, *in vivo*, and *ex vivo*

**DOI:** 10.1039/c9sc03368h

**Published:** 2019-08-16

**Authors:** Eunha Kim, Heebeom Koo

**Affiliations:** a Department of Molecular Science and Technology , Ajou University , Suwon 16499 , Republic of Korea; b Department of Medical Life Sciences , College of Medicine , The Catholic University of Korea , 222 Banpo-daero, Seocho-gu , Seoul , 06591 , Republic of Korea . Email: hbkoo@catholic.ac.kr; c Department of Biomedicine & Health Sciences , College of Medicine , The Catholic University of Korea , 222 Banpo-daero, Seocho-gu , Seoul , 06591 , Republic of Korea; d Catholic Photomedicine Research Institute , College of Medicine , The Catholic University of Korea , 222 Banpo-daero, Seocho-gu , Seoul , 06591 , Republic of Korea

## Abstract

Recently, click chemistry has provided important advances in biomedical research fields. Particularly, copper-free click chemistry including strain-promoted azide–alkyne cycloaddition (SPAAC) and inverse-electron-demand Diels–Alder (iEDDA) reactions enable fast and specific chemical conjugation under aqueous conditions without the need for toxic catalysts. Click chemistry has resulted in a change of paradigm, showing that artificial chemical reactions can occur on cell surfaces, in cell cytosol, or within the body, which is not easy with most other chemical reactions. Click chemistry *in vitro* allows specific labelling of cellular target proteins and studying of drug target engagement with drug surrogates in live cells. Furthermore, cellular membrane lipids and proteins could be selectively labelled with click chemistry *in vitro* and cells could be adhered together using click chemistry. Click chemistry *in vivo* enables efficient and effective molecular imaging and drug delivery for diagnosis and therapy. Click chemistry *ex vivo* can be used to develop molecular tools to understand tissue development, diagnosis of diseases, and therapeutic monitoring. Overall, the results from research to date suggest that click chemistry has emerged as a valuable tool in biomedical fields as well as in organic chemistry.

## Abstract 2

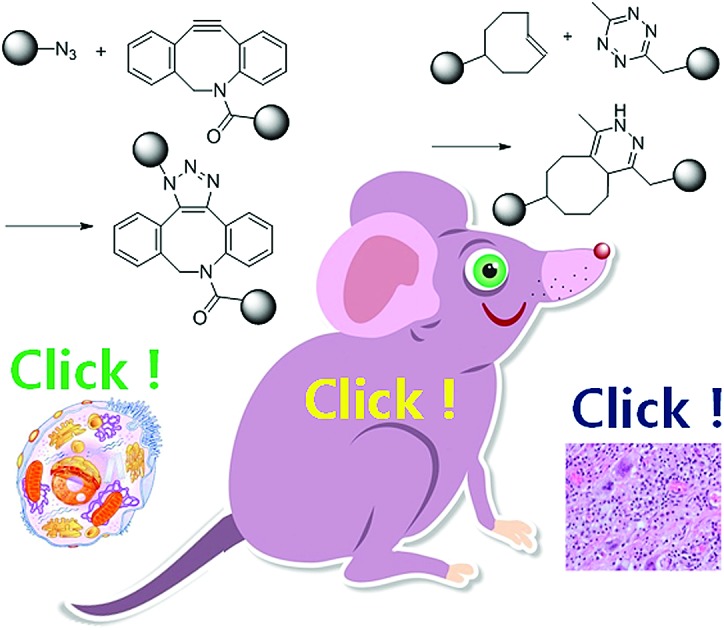
Copper-free click chemistry has resulted in a change of paradigm, showing that artificial chemical reactions can occur on cell surfaces, in cell cytosol, or within the body. It has emerged as a valuable tool in biomedical fields.

## 1. Introduction

Click chemistry has been broadly used for chemical reactions that have orthogonality, high yields, and fast kinetic second order reaction rate constants.^1^ These kinds of orthogonal reactions are useful for organic synthesis containing multiple steps and various functional groups. Most chemical reactions are not biocompatible because they require toxic catalysts, organic solvents, high temperatures, or high pressures.^2^ Consequently, all synthetic steps have previously been performed in flasks, on benches, or under fume hoods.

The resulting compounds can only be applied to a living cell or animal after complete purification. This means that spaces for carrying out artificial chemical reactions are completely segregated from biological spaces containing cells or animals. However, click chemistry is available under aqueous conditions with orthogonality, and offers potential for artificial chemical reactions being carried out on cell surfaces, in cell cytosol, or in the body.^3^ Particularly, organic chemists have attempted to remove the toxic copper catalyst from the representative click reaction, copper-catalyzed [3 + 2] azide–alkyne cycloaddition (CuAAC). Their trials resulted in ‘copper-free’ click chemistry which is highly attractive to biological or biomedical researchers.^4^–^6^

Initially, Staudinger ligation between azide and phosphine groups was the first chemical reaction thought to be bioorthogonal that could occur under aqueous conditions without use of a toxic catalyst.^7^

A few previous studies have introduced the application of this reaction on the cell surface or *in vivo*.^11^ However, this reaction is too slow to address researchers' requirements (the second order reaction rate constant was reported at approximately 7.7 × 10^–3^ M^–1^ s^–1^).^12^ Therefore, this reaction could not be considered as click chemistry.

K. Barry Sharpless first introduced the concept of click chemistry in 1998 when considering other reactions, and following this CuAAC has gained much attention.^1^ The second order reaction rate constant of CuAAC has been reported to be approximately 10 M^–1^ s^–1^ with 20 μM Cu(i), which is about 1000-fold faster than Staudinger ligation (Table 1).^13^ Another advantage of CuAAC is the small size of the functional groups involved in the reaction. Azide has only three nitrogen atoms while alkyne has one hydrogen and two carbons. Such a small size is helpful for minimizing perturbation of intrinsic characteristics of target molecules after modification. However, the cytotoxic copper catalyst restricts the *in vitro* or *in vivo* application of CuAAC reactions.

**Table 1:** Characteristics of currently used click chemistry reactions^*a*^

Name	Representative reaction	*k* (M^–1^ s^–1^)	Pros/cons
CuAAC	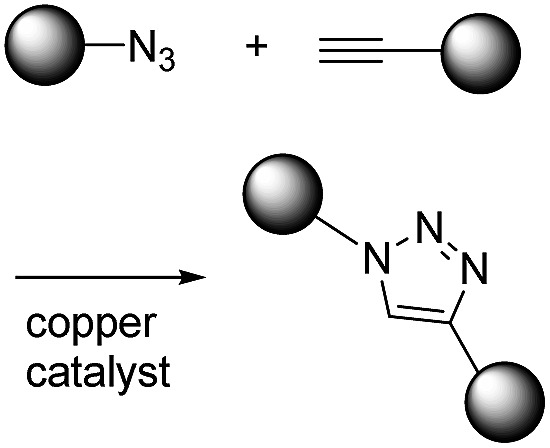	∼10–100 (with 20 μM Cu(i))^8^	- Both small structures
			- Cheap price
			- Toxic copper catalyst
			- High second order reaction rate constant
SPAAC	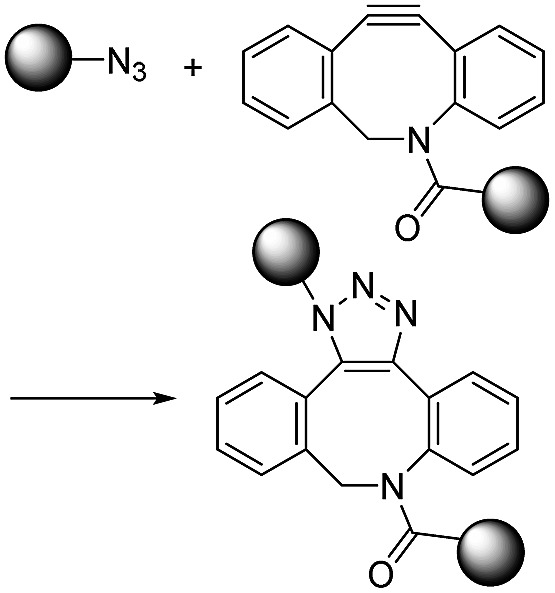	∼1–60 (M^–1^ s^–1^)^9^	- One small and one large structure
			- Moderate second order reaction rate constant
			- No catalyst
iEDDA	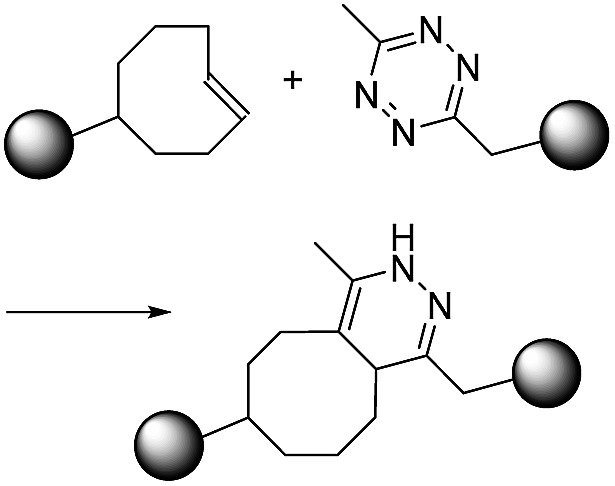	∼1–10^6^ (M^–1^ s^–1^)^10^	- Two large structures
			- Very high second order reaction rate constant
			- No catalyst

^*a*^CuAAC: copper-catalyzed [3 + 2] azide–alkyne cycloaddition; SPAAC: strain-promoted azide–alkyne cycloaddition; iEDDA: inverse-electron-demand Diels–Alder reaction.

To reduce the cytotoxicity of the copper catalyst in CuAAC, researchers tried to use stabilizing ligands. In 2010, Amo *et al.* used bis(*tert*-butyltriazoly) ligand which is water-soluble and effective in promoting CuAAC.^14^ Cells were alive after CuAAC using the ligand and it was applied to *in vivo* imaging of zebrafish embryo. Bevilacqua *et al.* and Kennedy *et al.* also demonstrated cell labelling by CuAAC using similar bis(*tert*-butyltriazoly) ligand and Cu(ii)–bis-l-histidine complex, respectively.^15^,^16^ To overcome this limitation fundamentally and increase the convenience, other chemists have increased the reactivity of alkynes using ring-strain which enables an azide–alkyne reaction without the need for a cytotoxic copper catalyst. These strain-promoted azide–alkyne cycloaddition reactions (SPAAC) have favourable second order reaction rate constants (approximately 0.1 M^–1^ s^–1^) under aqueous conditions without a catalyst.^17^

Dibenzocyclooctyne (DBCO) and bicyclo[6.1.0]nonyne (BCN) are representative of strained cyclic ring-containing alkynes for SPAAC. This copper-free click chemistry has combined chemical reactions with cell/animal studies.^18^ On the other hand, some chemists are not satisfied with the second order reaction rate constant of SPAAC and have attempted to find click reactions with second order reaction rate constants. They have found that inverse-electron-demand Diels–Alder (iEDDA) reactions between tetrazine (Tz) and *trans*-cyclooctene (TCO) could meet the requirements of click chemistry. This third-generation copper-free click chemistry provides a faster second order reaction rate constant (>10^3^ M^–1^ s^–1^) than CuAAC and SPAAC. Thus, it is considered a powerful tool for many purposes, further widening the field of click chemistry.^19^ The disadvantage of Tz–TCO click chemistry is that both molecules are somewhat larger than an azide or a normal alkyne. This large size can limit their application, as large click molecules can affect the intrinsic properties of target molecules after modification of the original molecules. Therefore, researchers need to consider the characteristics of click chemistry reactions carefully and select the most suitable reactions according to their intended purpose.

In particular, two different copper-free click chemistry reactions, SPAAC and iEDDA, allow bioorthogonal chemical conjugation in living organisms (on cell surfaces, in cell cytosol, or within the body). A number of reports have shown broad applications of click chemistry in the facile labelling of nucleotides or proteins, discovering unknown metabolic pathways, artificial adhesion of cells, spatiotemporal monitoring of newly grown tissues, surface-modification of nanoparticles, nanoparticle delivery, synthesis of hydrogels, and so on.^20^–^26^ These reports demonstrate that click chemistry is now an attractive technique in biomedical fields including imaging, drug delivery, or diagnostic analysis, as it is in organic chemistry.^27^,^28^ However, there are only a few papers focusing on recent applications of click chemistry in biomedical fields.^29^

In this review, we introduce recent research in which copper-free click chemistry is successfully applied in biomedical fields including imaging, drug delivery, and diagnostic analysis. We categorize this research based on the site at which the click reaction actually occurs, namely *in vitro*, *in vivo*, and *ex vivo* (Table 2). We conclude the review by highlighting precautions regarding click chemistry for biomedical purposes and the prospect of this technique in the future. Particularly, we will focus on copper-free click reactions occurring on a cell surface, in cell cytosol, in a body, or at least with proteins or nucleotides, emphasizing the advantage of copper-free click chemistry considering the environment in which the reaction occurs. Click chemistry also contributes to the development of drugs or materials for biomedical applications. However, we did not cover these kinds of applications of click chemistry outside of cells or animals in the present paper because they have been summarized well in other reviews.^30^,^31^

**Table 2:** Summary of the copper-free click chemistry research for biomedical applications

Location	Application	Click molecules used	Type
*In vitro*	PARP1 imaging^35^	AZD2281-TCO/Tz-Texas Red	iEDDA
	PARP1 target identification study^36^	AZD2281-TCO/Tz-cleavable linker-biotin	iEDDA
	Tubulin protein imaging^37^	Taxol-TCO/Tz-BODIPY FL	iEDDA
	Polo-like kinase 1 protein imaging^38^	BI2536-TCO/Tz-Texas Red	iEDDA
	Aurora kinase A protein imaging^39^	MLN8054-TCO/Tz-CFDA, Tz-Texas Red	iEDDA
	MET protein imaging^40^	PF04217903-TCO/Tz-CFDA	iEDDA
		Foretinib-TCO/Tz-CFDA	iEDDA
	Imaging binding targets of drug molecules^41^	Dasatinib-TCO/Tz-CFDA	iEDDA
	BTK protein imaging^42^	Ibrutinib-TCO/Tz-Cy5	iEDDA, SPACC
		Ibrutinib-N_3_/DBCO-Cy5	
	Drug imaging^43^	JQ1-TCO/Tz-Cy5	iEDDA
	Visualization of PTM modification^44^	NAD^+^-DBCO/N_3_-Cy3, N_3_-Cy5	SPACC
		NAD^+^-methyl cyclopropene/Tz-Cy3	iEDDA
	Site-specific oligonucleotide labelling^45^–^47^	RNA-norbornene/Tz-Oregon Green 488	iEDDA
		RNA-methyl cyclopropene/Tz-Oregon Green 488	
		DNA-Tz/BCN-rhodamine	
		DNA-methyl cyclopropene/Tz-BODIPY	
	Site-specific protein labelling^48^	l-Lysine-TCO/Tz-Cy5	iEDDA
	No-wash STED imaging^49^	Phalloidin-BCN/Tz-Cy3	iEDDA
*In vivo*	Zebrafish embryo growth imaging^24^	*N*-Acetylgalactosamine (GalNAc)/difluorinated cyclooctyne (DIFO)-Alexa Fluor dye	SPACC
	Glycan imaging by intraperitoneal injection^50^	Tetraacetylated *N*-azidoacetyl-d-mannosamine (Ac_4_ManNAz)/DIFO-FLAG peptide conjugate	SPACC
	Brain glycan imaging^51^	Liposomes loading 9-azido sialic acid/DBCO-Cy5.5	SPACC
	Tumor glycan imaging by SPECT^52^	Anti-TAG72 antibody CC49-TCO/Tz-^111^In	SPACC
	Tumor glycan imaging by MR^53^	Ac_4_GalNAz/TMDIBO-Lys-Gd (tetramethoxydibenzocyclooctyne-lysine-gadolinium)	SPACC
	Tumor VEGF (vascular endothelial growth factor receptor 2) imaging by ultrasound^54^	Antivascular endothelial growth factor receptor 2 antibody (TCO-antiVEGFR2)/Tz-modified microbubbles	iEDDA
	Acute thrombus imaging by ultrasound^55^	AntiCD62p-TCO/Tz-modified microbubbles	iEDDA
	Tumor-targeted liposomes for drug delivery^25^	Ac_4_ManNAz/DBCO-modified liposome	SPACC
	Tumor-targeted photosensitizer delivery^27^	Ac_4_ManNAz-loading chitosan nanoparticles/DBCO-modified chitosan nanoparticles	SPACC
	Tumor-targeted doxorubicin delivery^56^	Peptide-modified Ac_4_ManNAz/DBCO-modified doxorubicin	SPACC
	Upconversion nanoparticle-based photodynamic therapy^57^	Upconversion NP modified with Tz groups/norborane (NB)-conjugated rose bengal (RB)	iEDDA
	Endothelial progenitor cell therapy for myocardiac infarction^58^	Azide-modified CD34 antibody/DBCO-modified CD41 antibody	SPACC
*Ex vivo*	Glycoproteomic analysis of intact and hypertrophy hearts^59^	Ac_4_ManNAz/DBCO-Fluor 488	SPACC
	Analysis of microvesicles from the blood of glioblastomas patients^60^	Antibody-modified TCO/Tz-modified magnetic nanoparticles	iEDDA
	Amplification of signal for diagnosis purpose^61^	Antibody-modified TCO/Tz-modified magneto-fluorescent nanoparticles	iEDDA
	Study of protein dynamics in the neuronal system^62^	Azidohomoalanine/DIFO-biotin	SPACC
	Study of the conformational change of protein by FRET^63^	Unnatural amino acid containing tetrazine or norbornene/BODIPY-FL tetrazine or BODIPY-TMR-X bicyclononyne	iEDDA, SPACC
	Nucleotide ligation for the CRISPR Cas9 system^64^	RNAs modified with alkyne or DBCO/RNAs modified with azide	CuAAC, SPACC

In addition, ‘photoclick’ reactions between electron-deficient olefins and diaryltetrazoles are also an attractive tool with special advantages.^32^ These reactions can be triggered by light of a particular wavelength, which means that two molecules are conjugated to each other under spatiotemporal control depending on the purpose. Furthermore, the nitrilimine produced by photoexcitation of diaryltetrazole is highly reactive, so that the reaction rate is very fast (*k* ∼ 60 M^–1^ s^–1^).^33^ A recent study of the Wagenknecht group used this reaction for facile labelling of DNA using cyanine dye showing the utility of the photoclick reaction.^34^ However, the wavelength of light used in photoclick reactions is generally in the ultraviolet (UV) range, which is a limitation in biomedical research. It is well-known that UV light is cytotoxic, so it is not suitable for cell studies. In addition, its short wavelength resulted in poor tissue penetration for application *in vivo*. For these reasons, it has been applied mainly for research outside of cells or animals. If a new photoclick reaction using visible or NIR light is proposed in the future, it will be more useful for biomedical applications.

## 2. Click chemistry *in vitro*

### 2.1 Clickable drug surrogates

One of the most interesting applications of copper-free click chemistry might be the fluorescence imaging of target of interest (TOI) proteins inside cells. The small molecular size of the tag for click chemistry means that a known ligand could be modified with the tag for labelling TOI proteins with minimized reduction of their original binding while retaining their cell permeability. Especially with iEDDA reactions, innate TOI proteins in live cells could be successfully visualized with a TCO–ligand conjugate and subsequent treatment of Tz containing fluorophores (FL_Tz_).

**Fig. 1:**
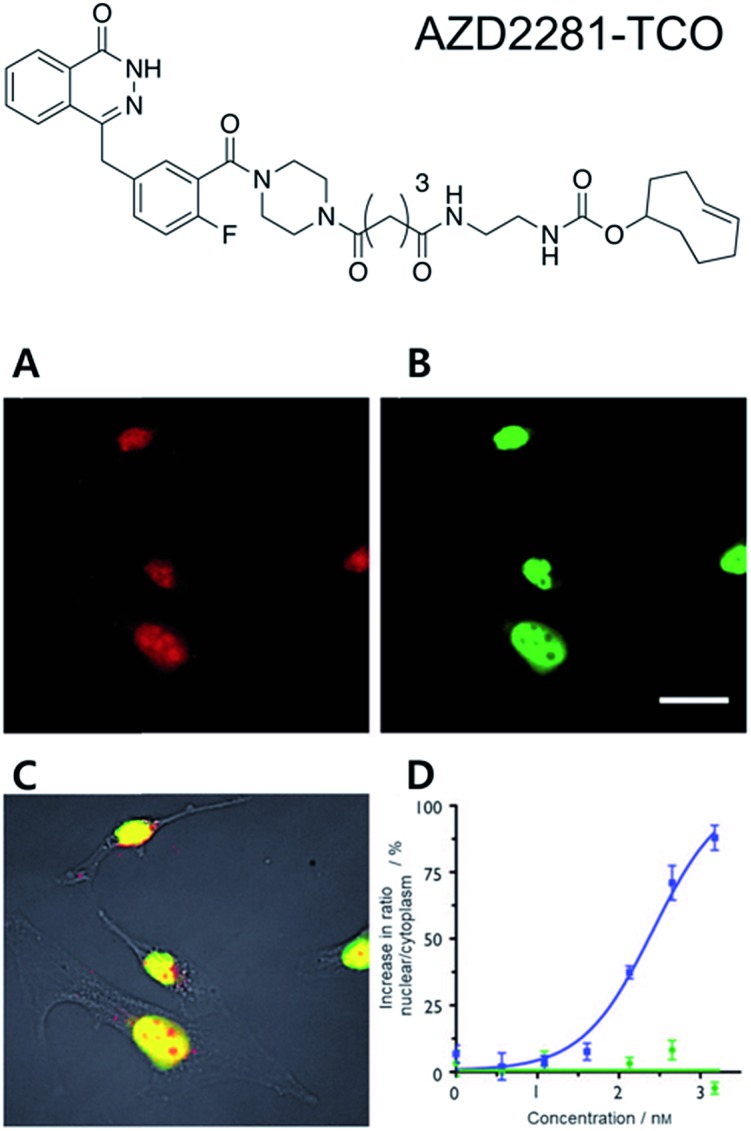
Clickable drug surrogates for imaging of PARP1 protein *in vitro*. (A) Copper-free click reaction between AZD2281-TCO and Texas Red-Tz in MDA-MB436 cells. (B) Immunofluorescence staining of PARP1 protein with green fluorescent monoclonal anti-PARP antibody. (C) A composite overlay on phase contrast. (D) Increase in the ratio of nuclear/cytoplasmic fluorescence signal for AZD2291-TCO/Texas Red-Tz without (circle) and with (square) the blocking reagent AZD2281. Scale bar: 20 μm. Reproduced from ref. 35 with permission from John Wiley and Sons, copyright 2010.

A proof of concept study for this strategy was reported by the Weissleder group in 2010.^35^ In that study, they conjugated the clinical drug AZD2281 with TCO to develop a bio-probe for studying poly(ADP-ribose) polymerase-1 (PARP1) proteins which are known to be important cellular proteins for DNA repair (Fig. 1). They found that TCO modification of AZD2281 resulted in marginal IC_50_ change from 5 nM to 11 nM. In addition, they found that the AZD2281-TCO conjugate was localized to the target PARP protein in the cell with Texas Red-Tz. This localization was inhibited by the original AZD2281 compound. Similar results were obtained for other clinical drugs. For example, they conjugated TCO to the well-known anticancer agent, Taxol, and successfully visualized tubulin proteins inside cells with a Taxol-TCO/Tz-BODIPY FL combination.^37^ After successful demonstrations, multiple ligand–TCO conjugates such as BI2536,^38^ MLN8054,^39^ PF04217903,^40^ Foretinib,^40^ Dasatinib,^41^ and Ibrutinib^42^ have been developed for targeting various TOI proteins such as polo-like kinase 1 (PLK1), Aurora kinase A (AURKA), MET, ABL1, SRC, CSK and Bruton's tyrosine kinase (BTK) proteins.

Click chemistry is useful not only for imaging cellular proteins but also for studying drug target engagement. Many drug candidates often fail to be considered clinically viable due to undesirable interactions with multiple different side targets. To better understand actual target proteins and pharmacokinetics *in vitro*, click chemistry has been highlighted as a powerful chemical tool for target identification and drug-target engagement studies in *in vitro* systems. For example, the Weissleder group has reported on bioorthogonal proteomics using copper-free click chemistry.^36^ They modified Olaparib with TCO for target identification of the drug. In addition, they further developed a cleavable enrichment linker containing Tz (for click chemistry), biotin (for pull down assay), and 2-(4′-hydroxy-2′-alkoxy phenylazo)benzoic acid (as a cleavable site). They first confirmed the activity of Olaparib-TCO against recombinant PARP1 proteins and confirmed that Olaparib-TCO still had a nano-molar range of IC_50_. Later, MHH-ES1 Ewing's sarcoma cells and A2780 ovarian cancer cells were treated with Olaparib-TCO. Proteins labeled with TCO drugs were then pulled down by two-step bioorthogonal magnetic separation. To release proteins from magnetic beads, sodium dithionite was used to cleave the linker that allowed the specific release of small-molecule captured proteins while leaving non-specifically bound proteins on the solid support. Interestingly, LC/MS-MS data revealed a list of 24 different proteins including PARP1 proteins. During a follow-up in-depth study, they identified the protein TOP2A as another binding partner of Olaparib with an estimated *K*_d_ of 3.7 nM.

**Fig. 2:**
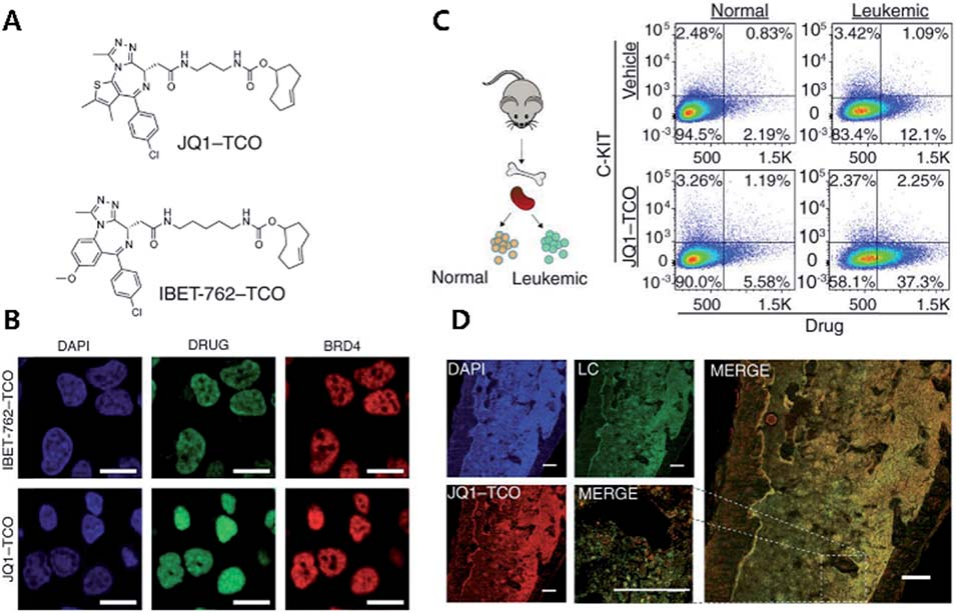
Clickable drug surrogates for mechanism study *in vitro*. (A) Chemical structures of clickable BET inhibitor analogs, JQ1-TCO and IBET-762-TCO. (B) Fluorescence imaging of HeLa cells incubated with IBET-762-TCO or JQ1-TCO. Nuclei were stained with 4′,6-diamidino-2-phenylindole (DAPI), drug surrogates were labelled with Cy5-tetrazine, and BRD4 was stained with an anti-BRD4 antibody. Scale bar: 20 μm. (C) Schematic illustration of the procedure to detect clickable small molecules *in vivo* and flow cytometry analysis of drug levels within normal hematopoietic cells and leukemia cells. (D) Confocal microscopy of individual leukemia cells containing drug surrogates in mouse femur tissue treated with 100 mg kg^–1^ of JQ1-TCO. Leukemia cells (LCs) are identified by the Venus reporter. Scale bar: 187 μm. Reproduced from ref. 43 with permission from The American Association for the Advancement of Science, copyright 2017.

A recent mechanism study of BET bromodomain inhibitors and epigenetic-based therapy has highlighted the potential of click chemistry for pre-clinical assessment of various drugs (Fig. 2).^43^

In that study, the authors synthesized derivatives of BET inhibitors such as JQ1 and IBET-762 compounds with propargyl and TCO for copper-catalyzed and iEDDA type click chemistry. They first confirmed that modification of the original drug molecules with a click chemistry functional group did not influence the functional integrity of BET inhibitors using cell-based assay, ChIP-qPCR assay, and RNA-Seq. Subsequently, they revealed, with JQ1-TCO, differences in the binding modes of BRD4 between BET inhibitor responsive and unresponsive genes. They found that bromodomain 1 (BD1) could mediate the binding of BRD4 proteins to acetyl-lysine sites and that bromodomain 2 (BD2) was the binding site of clickable drug surrogates. Furthermore, they evaluated the pharmacokinetics of the parent drug with clickable drug surrogates and confirmed the heterogeneity of drug distribution between organs as well as between different cell types in the same organ. These examples highlight the power of copper-free click chemistry for studying actual targets, pharmacokinetics (PK), and pharmacodynamics (PD) of drug molecules.

Click chemistry can be used to address long-standing mechanistic questions in the clinic. For example, the Nathan group reported the traceable mimic of the anti-cancer drug cytarabine (ara-C) by converting a single hydroxyl group to azide, giving AzC. They confirmed the equal biological profile of AzC in cell culture and *in vivo* as ara-C. Further studies of the AzC with classical azide–alkyne click chemistry, including the gSTED imaging experiment, allowed them to understand the contradiction of cell-type selectivity of nucleoside-based drugs.^65^

### 2.2 Click chemistry for labelling cellular membrane lipids and proteins

**Fig. 3:**
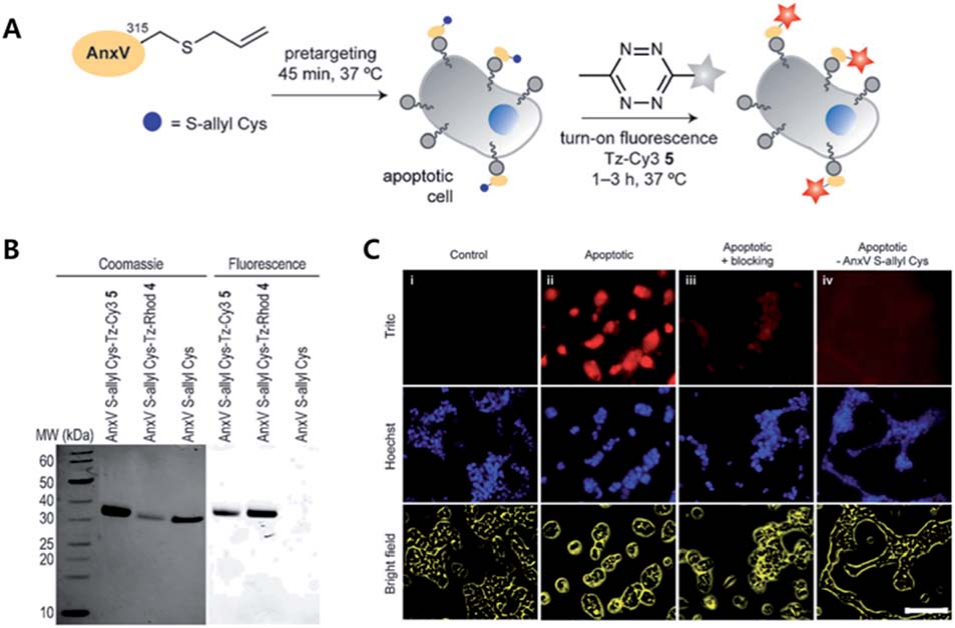
Click chemistry for labelling cellular membrane protein. (A) *In vitro* bioorthogonal labelling of apoptotic cells with an *S*-allyl handle by iEDDA copper-free click chemistry. (B) SDS-PAGE of AnxV containing an *S*-allyl handle labeled with Tz-Cy3 or Tz-Rhod. Fluorescence scanning of gels imaged (right) before staining with Coomassie blue (left). (C) *In vitro* fluorescence imaging of apoptotic cells with AnxV containing a chemical handle for the iEDDA reaction. Apoptotic cells were specifically labeled with AnxV *S*-allyl Cys incubation and followed by treatment with fluorogenic Tz-Cy3. Scale bar: 100 μm. Reproduced from ref. 66 with permission from John Wiley and Sons, copyright 2016.

To avoid issues with Tz or TCO such as metabolic stability, cross-reactivity with biological nucleophiles, and impact on protein structure, activity, or localization, tags with smaller sizes than to conventional iEDDA tags have been suggested. For example, Oliveira *et al.* reported that unstrained *S*-allyl could be used as the tag for iEDDA type bioorthogonal labelling under live cell conditions (Fig. 3).^66^ They first surveyed the reaction kinetics of *S*-allyl cysteine with PyTz, BnNH_2_-Tz, Tz-Rhod, and Tz-Cy3 in a PBS (pH 7.4) and methanol mixture (v/v = 1 : 1) at 37 °C. They found that *S*-allyl Cys had reasonable reaction kinetics for iEDDA reactions with screened Tzs (*k*^2^ from 2.05 × 10^–3^ to 0.26 × 10^–3^ M^–1^ s^–1^), which was comparable to SPAAC reaction kinetics (10^–1^*k*^2^ = 10^–3^ M^–1^ s^–1^). In addition, this chemistry allowed them to achieve specific recognition of apoptotic cells *via* [2,3]-sigmatropic rearrangement of allyl selenocyanate with phosphatidyl serine (PS) membrane lipids on the cellular surface. With Annexin V and engineered variants of the C2A domain of synaptotagmin-I (C2Am), they confirmed more efficient *S*-allylation of Cys *via* [2,3]-sigmatropic rearrangement than with direct allylation. Due to the fluorogenic properties of Tz-Cy3, they successfully imaged apoptotic cells without the washing step.

To develop light-gated ion channels with longer excitation wavelengths, simple experimental setup, and efficient activation, the Xing group has incorporated NIR-sensitive-UCNs (upconversion nanocrystals) with light-gated channelrhodopsin-2 proteins (ChR2) *via* a copper-free click reaction between the DBCO (dibenzyl cyclooctene) moiety on UCNs and an azido tag on membrane proteins.^67^ Firstly, they confirmed the azide group labelling of membrane glycans in HEK293 (human embryonic kidney 293) cells based on metabolic glycoengineering by feeding tetraacetylated *N*-azidoacetyl-d-mannosamine (Ac_4_ManNAz), following covalent labelling with a DBCO-Cy3 fluorophore. Later, ChR2 proteins were engineered to be expressed on the cell surface and their cellular location was confirmed with GFP markers on the proteins. UCNs doped with Nd^3+^ were functionalized with polyacrylic acid (PAA) while DBCO moieties were coupled with a carboxylic group in PAA. Coating of DBCO on the surface of UCNs was confirmed with click reactions using FAM-N_3_ (5-carboxyfluorescein-azide). Then, they confirmed specific labelling of ChR2 proteins on the HEK293 cell membrane with UCNs using copper-free click chemistry. Influx of Ca^2+^ ions into cells was triggered by 808 nm excitation and Ca^2+^-dependent apoptosis was confirmed using Cy3-tagged Annexin V. Finally, they implanted N_3_-tagged HEK292 cells expressing ChR2 proteins into the yolk sac of zebrafish and incubated the larvae with DBCO/Cy5.5-UCNs. They demonstrated successful manipulation of Ca^2+^ influx *in vivo* using copper-free click chemistry-mediated site-specific labelling and light activation of Ca^2+^ channel proteins.

Non-canonical amino acids (ncAAs) approach is one of the most versatile methods for labeling in a residue specific fashion for the TOI protein and recent advances in the techniques allowed site-specific incorporation of strained alkene and alkyne to the TOI protein. In 2016, Lemke group reported in cell protein labeling utilizing cyclooctyne and *trans*-cyclooctene amino acid by utilizing the iEDDA reaction with 1,2,4,5-tetrazine.^48^ First, they found NLS (nuclear localization signal) on *M. mazei* PylRS protein sequence. They found nuclear localization of PylRSAF protein in HEK293T and COS-7 cells with IF (immunofluorescence) staining and FISH (fluorescence *in situ* hybridization) study. By cytoplasmic localization reinforcement of the protein *via* addition of a nuclear export signal (NES), they improved the efficiency of amber suppression with the engineered protein (NESPylRSAF). With transcription factor jun-B, they confirmed that NESPylRSAF successfully incorporated ({[(*E*)-cyclooct-2-ene-1-yl]oxy}carbonyl)-l-lysine (TCO*a) with a better signal to noise ratio than PylRSAF. They further applied the system for super resolution microscopy (SRM) with a “Click-PAINT” approach. Basically, first the TOITAG → TCO*a protein was equipped with Tz-ssDNA (the “docking strand”), then it was labeled with a complementary ssDNA (the “imaging strand”) containing a photostable synthetic dye. They applied this technique to image Nup153 protein consisting of a nuclear pore complex (NPC) and successfully visualized the detailed structure of NPC. This study highlights that the combination of the iEDDA reaction with other biotechniques, such as DNA-PAINT, could provide more powerful fluorescence imaging techniques for visualizing TOI protein to allow a more in-depth study of biological systems.

A recent study by Marx group highlights the potential of copper-free click chemistry not only for simple labeling of the protein but also for the visualization of post-translational modification (PTM) and cellular signaling in the cells.^44^ In this study, they developed functionalized NAD^+^ analogues to study poly(ADP-ribos)ylation (PARylation) in the cells as a traceable substrate for ARTD1, one of the most well studied ADP-ribosyltransferases (ARTs). By modifying adenosine with a terminal alkyne, terminal alkene, azide, DBCO and cyclopropene they developed 10 different clickable probes as substrates for ARTD1. The screening result confirmed that 2-modified NADs have good tolerance against ARTD1. Finally, they achieved bioorthogonal labeling of PAR in multiple fluorescence channels *via* SPAAC and iEDDA reactions in the cells, incubated with two different functionalized NAD^+^ analogues, between the analogues and clickable fluorescent dye such as Cy5-N_3_ or Cy3-Tz, respectively.

### 2.3 Cell adhesion

Recent studies on CAR T cell therapy and the effects of macrophages on tumor microenvironment have indicated that cell–cell interactions are emerging as some of the most promising therapeutic strategies.^68^,^69^ Artificial cell adhesion could provide a valuable chemical tool box for therapeutic applications. Multiple attempts using nucleotides, avidin–biotin interactions, antibody dimers, and aptamers have been performed under *in vitro* conditions.^23^,^70^,^71^ However, due to the possible degradation of the adhesive for cell–cell interactions by enzymes, such as proteases, DNases, and RNases, *in vivo* conditions are a drawback of complementary macromolecules.^72^ Therefore, bioorthogonal small molecules could provide alternative approaches for artificial cell adhesion with more stable, robust, multivalent, and economical chemical bonds.

**Fig. 4:**
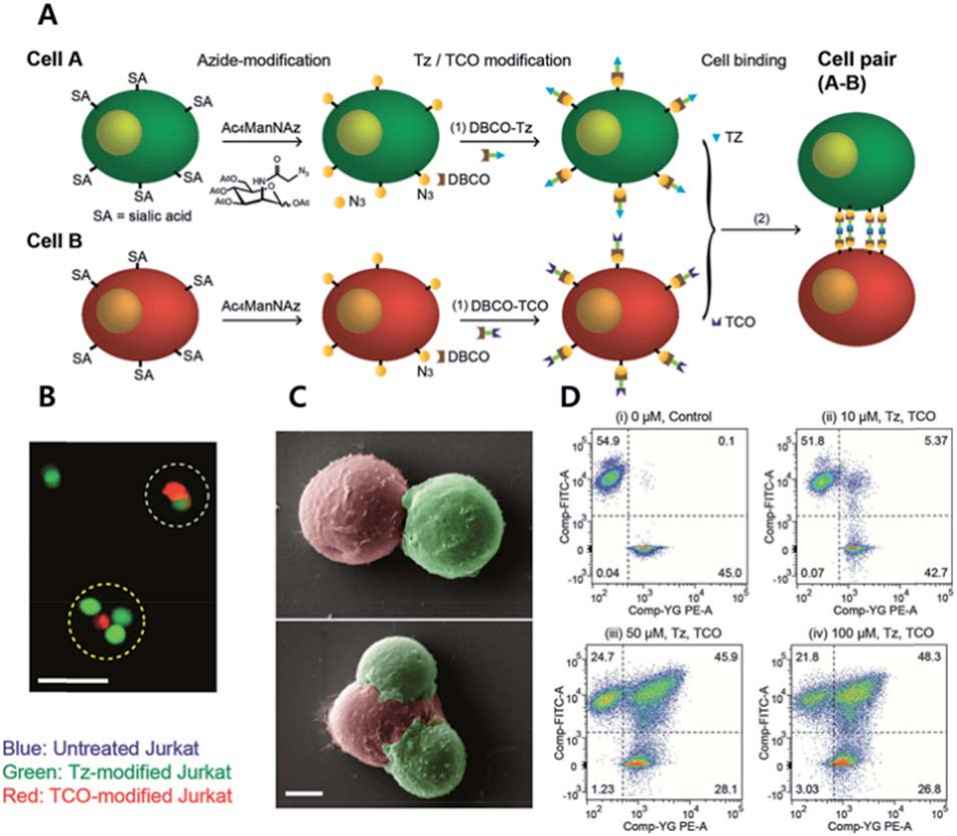
Click chemistry for artificial cell attachment. (A) Schematic illustration of the artificial cellular gluing method *via* metabolic glycoengineering and copper-free click chemistry. (B) Fluorescence images and (C) SEM images of glued cells visualized doublet to quartet glued cells. Scale bar: 50 μm in (B) and 5 μm in (C). (D) Flow cytometry data of glued cells confirmed dose-dependent population increase of glued cells. Reproduced from ref. 73 with permission from John Wiley and Sons, copyright 2015.

Koo *et al.* have studied TCO/Tz mediated cell adhesion (Fig. 4).^73^ Cells including A549 human lung cancer, Jurkat T cell lymphoma, NIH3T3 murine fibroblasts, and EL4 lymphoma cells were first tagged with an azido group using metabolic glycoengineering with Ac_4_ManNAz. Subsequently, Tz and TCO were incorporated onto azido-modified sialic acid by Tz-DBCO or TCO-DBCO treatment. After TCO modified Jurkat T cells were added to layers of TCO modified A549 cells in a microfluidic setting, it was confirmed that artificial adhesion between A549 and Jurkat T cells was completed within 10 min. In addition, adhered cells displayed stable interactions in microfluidic channels even under a flow rate of 60 mL min^–1^ with a calculated shear stress of 20 dyn cm^2^ (2 Pa). After that, adhered cell pairs were introduced to wild-type mice by retro-orbital injection. Adhered cells were present in murine blood vessels, they were found using two-photon microscopy. In addition, intravenously injected cell pairs were localized to lung tissues. They were trapped in lung capillary beds while maintaining their attachment, even under *in vivo* conditions. A recent study by the same group has demonstrated that adhered cells could be detached by chemical stimuli.^74^ They first introduced an azido group onto Jurkat and A549 cells *via* metabolic glycoengineering using Ac_4_ManNAz. Later, azide-modified A549 and Jurkat cells reacted with DBCO-disulfide-Tz and DBCO-disulfide-TCO, respectively. They confirmed that treatment with 5 mM glutathione reduced the ratio of attached Jurkat cells onto A549 cells down to 10% due to cleavage of disulfide bonds.^75^

### 2.4 Tetrazine-based turn-on probe

One of the most interesting features of Tz, especially for fluorescence imaging, is the dual functionality of the compound. It can act as a bioorthogonal reaction unit and as a fluorescence quencher simultaneously.^37^,^76^–^79^ Therefore, fluorophores conjugated with Tz could be used as bioorthogonal reaction turn-on probes for washing-free fluorescent imaging. Starting with the BODIPY FL fluorophore, multiple different fluorophore-Tz conjugates (FL_Tz_) have been developed. Most of the quenching mechanisms of FL_Tz_ are based on energy transfer, which is basically the transfer of energy from excited fluorophores to Tz units. The energy is released in a non-radiative manner.

Two different energy transfer mechanisms are known. One type is based on long-range dipole–dipole interactions and is known as Förster resonance energy transfer (FRET). The other is a through-bond energy transfer (TBET) mechanism induced by making the FL_Tz_ a conjugated, but not coplanar, pi-system (resulting in a twist in the inter-chromophore linkage). The first usage of FL_Tz_ for bioorthogonal turn-on probes was reported with the FRET type mechanism.^37^ In that study, the authors conjugated 3-(4-benzylamino)-1,2,4,5-tetrazine with BODIPY FL, Oregon Green 488, and BODIPY TMR-X. All FL_Tz_s in that study exhibited turn-on effects after reaction with TCO. The turn-on efficiency increased from 1-fold to 20-fold. Soon after, the TBET based strategy was reported.^80^ Instead of a simple coupling between Tz and fluorophores, the authors directly connected Tz with the BODIPY fluorophores. Using this strategy, the authors developed a bioorthogonal turn-on probe exhibiting increased fluorescence up to 1600-fold. They also successfully applied these probes to washing-free fluorescence imaging of EGFR proteins with TCO modified antibodies.

**Fig. 5:**
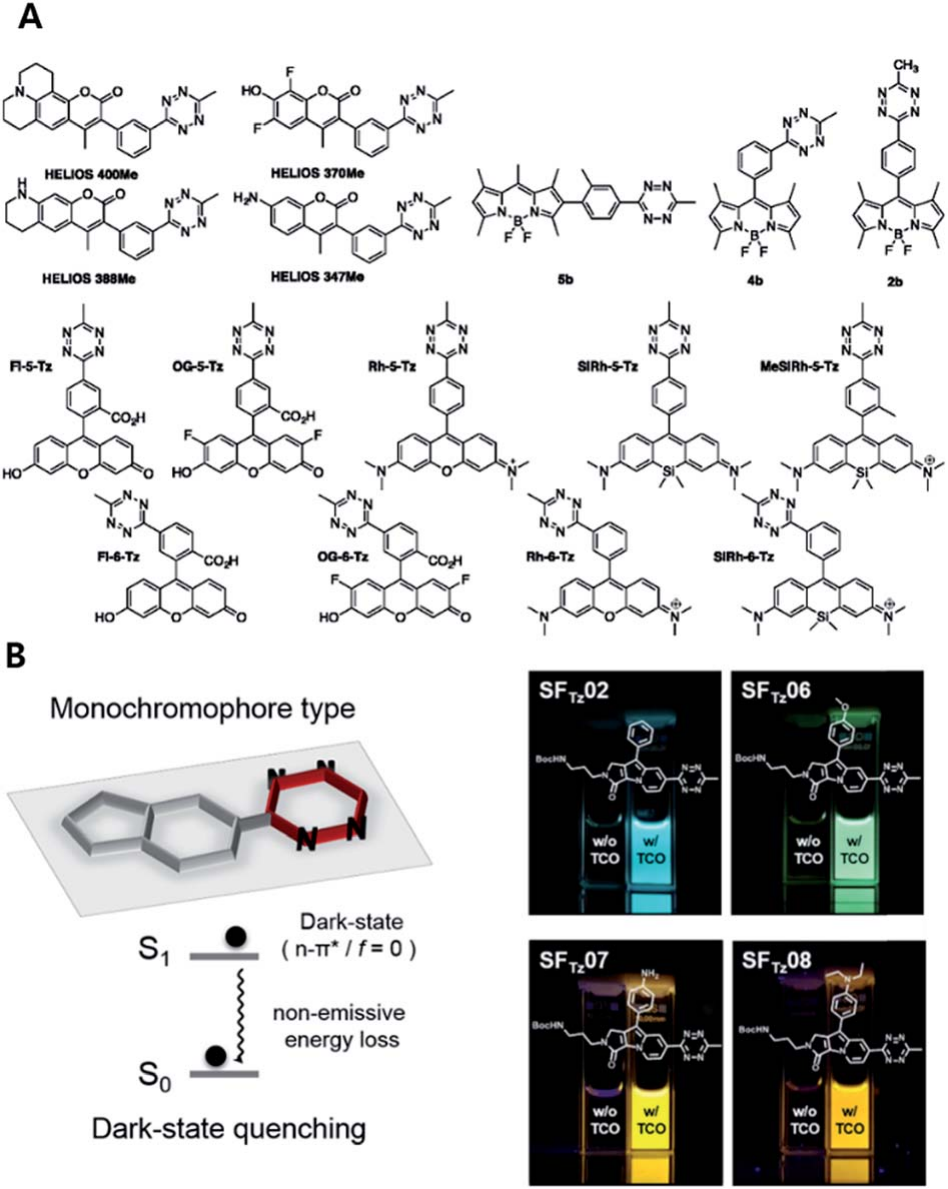
Tetrazine-based turn on probe development. (A) Chemical structures of fluorogenic tetrazine fluorophores (FL_Tz_). (B) Monochromophore type FL_Tz_ allows wavelength independent fluorescence quenching efficiency *via* nonradiative energy decay due to the lowest lysing dark state at S1 presumably originated from a non-radiative n → π* transition. Reproduced from ref. 86 with permission from American Chemical Society, copyright 2017.

Hitherto, coumarin, BODIPY, fluorescein, rhodamine, phenoxazine and silicon rhodamine fluorophores have been utilized to develop FL_Tz_ bioorthogonal turn-on probes.^81^–^85^ A drawback of previous energy transfer type quenching mechanisms is the reduced quenching efficiency against more red-shifted fluorophores. The energy accepting efficiency of the energy acceptor Tz is approximately up to 500 nm, therefore fluorophores with longer emission wavelengths exhibit a lesser turn-on effect. To overcome limitations with previous approaches, a monochromophoric design strategy was recently reported to develop bioorthogonal turn-on probes regardless of their color (Fig. 5).^86^ In that study, Lee *et al.* introduced a new molecular design approach for full integration between Tz and a model fluorophore system. They confirmed that fluorophores under the new molecular design approach exhibited increased turn-on efficiency by 600- to 1000-fold, regardless of the emission wavelength of the fluorophore.

The fluorogenic FL_Tz_ probe is also useful for super resolution microscopy (SRM) imaging.^79^,^87^–^90^ In 2018 the Kele group reported fluorogenic cyanine-tetrazine conjugates for no-wash subsequent super-resolution microscopy (STED) imaging of an actin filament. To quench the cyanine dye *via* the TBET strategy along with bioorthogonality, they designed 4 different tetrazine-cyanine dyes utilizing either a phenylene or a vinylene linker between tetrazine and the fluorophore. They found that all four probes showed fluorogenic behavior but found 14 fold fluorescence enhancement by conjugating Tz on Cy3 with the vinylene linker and the other 3 different probes exhibited 3 fold fluorescence enhancement in average. With phalloidin-BCN, they successfully applied the probe for STED imaging of actin filaments with 168 nm resolution using a 660 nm continuous wave laser for depletion.^49^

## 3. Click chemistry *in vivo*

### 3.1 *In vivo* imaging

Artificial chemical reactions *in vivo* have been challenging for researchers because the environment in a living body has large amounts of different molecules including ions, small chemicals, nucleotides, and proteins. This means that bioorthogonality is required for reactions *in vivo*. Furthermore, the concentration and contact time of click molecules are limited *in vivo*, and the second order reaction rate constant is also important.

In 2008, the Bertozzi group introduced an excellent application of click chemistry *in vivo* for spatiotemporal imaging of zebrafish embryo growth.^24^ In that study, the authors treated zebrafish embryos with *N*-acetylgalactosamine (GalNAc) to modify glycan with azide groups by metabolic glycoengineering. They then applied difluorinated cyclooctyne (DIFO)-Alexa Fluor dye that could be conjugated with azide groups by SPAAC. They repeated this process with similar dye conjugates of different colors at predetermined time points. Finally, they obtained adult zebrafish labeled with multiple colors of fluorescence. These labeled colors demonstrated the time points at which different sites developed during growth. These images effectively provided spatiotemporal information of zebrafish growth.

In 2010, the same authors further demonstrated that SPAAC *in vivo* could be applied to mice.^50^ They injected Ac_4_ManNAz into mice by intraperitoneal injection for azide group incorporation by metabolic glycoengineering. They then injected various molecules that could bind azide groups similarly. Based on the analysis of excised splenocytes from the mice, they determined the conjugation efficacy between azide groups and different chemicals. Their results showed that dimethoxy azacyclooctyne (DIMAC) had the highest efficacy among the ring-strained alkynes used. They also found that the hydrophobicity of chemicals induced aggregation with serum proteins resulting in non-specific binding to other tissues. Interestingly, in their paper, Staudinger ligation was efficient, suggesting that it was even better than ring-strained alkynes. The intraperitoneal space represents an environment with special conditions. It is without active in- or out-flow compared to intravenous (i.v.) injections, which is helpful for maintaining sufficient concentrations of reactive molecules while minimizing the effects of reaction rate between them.

**Fig. 6:**
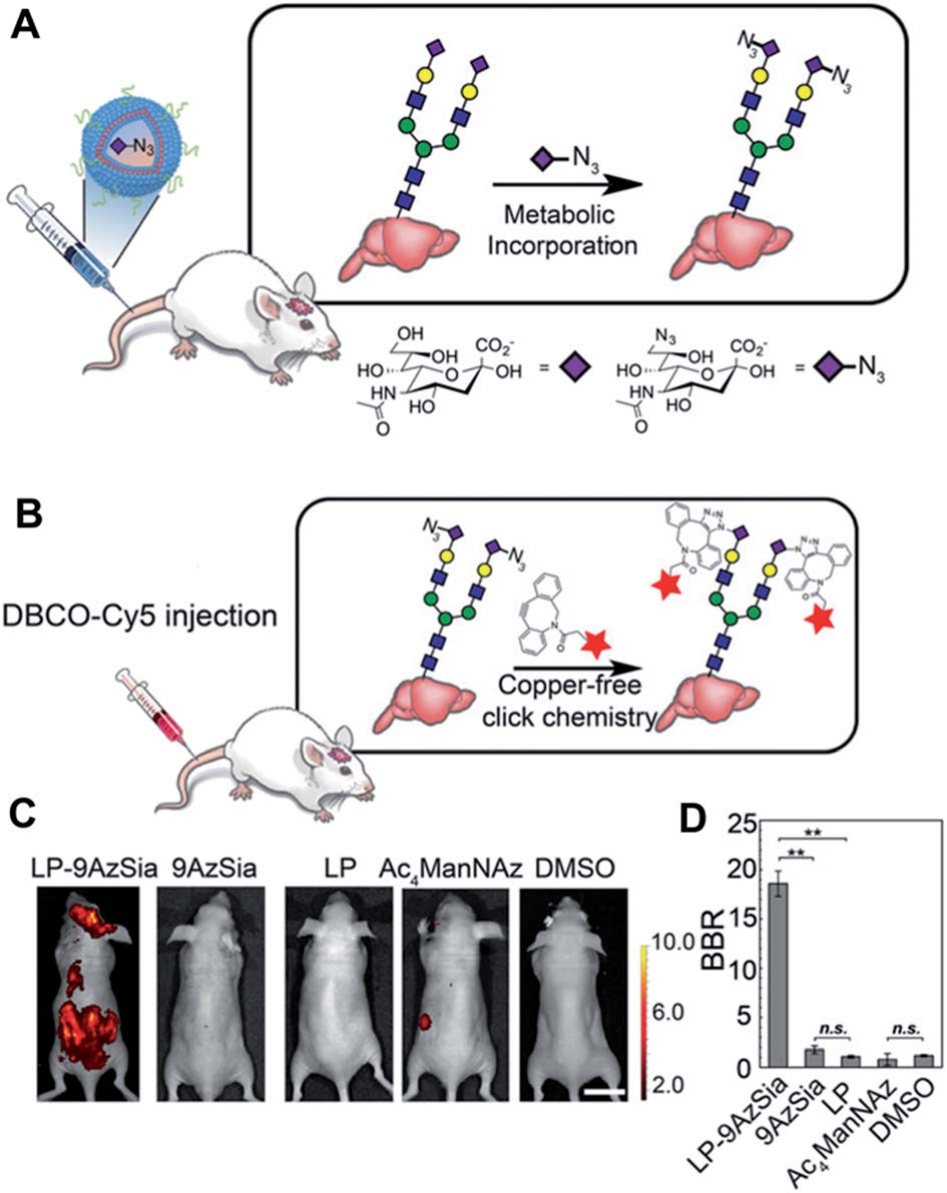
*In vivo* fluorescence imaging of sialoglycans in mouse brain by copper-free click chemistry. (A) Azide group labelling of sialoglycans in mouse brain by 9-azido sialic acid-loaded liposome and metabolic glycoengineering. Liposomes were injected intravenously to mice and delivered 9-azido sialic acid to brain cells across BBB. Then, azide groups were generated in brain cells by metabolic glycoengineering. (B) SPAAC *in vivo* between DBCO-Cy5 and sialoglycans in mouse brain. Intravenously injected DBCO-Cy5 could label newly synthesized brain sialoglycans. (C) *In vivo* fluorescence images showing sialoglycans targeted by SPAAC. 9-Azido sialic acid-loaded liposomes were administered daily to mice for 7 days and DBCO-Cy5 was injected at day 8. (D) Brain signal-to-background ratio (BBR) of (C). **P < 0.01; n.s., not significant (one-way ANOVA). Reproduced from ref. 51 with permission from the National Academy of Sciences of the United States of America (NAS), copyright 2016.

Encouraged by these promising results regarding click chemistry *in vivo*, researchers have attempted to utilize click chemistry to obtain biological or biomedical information from mice. Recently, Xie *et al.* introduced *in vivo* labelling of brain sialoglycans using liposomes (Fig. 6).^51^ 9-Azido sialic acid, a metabolic precursor used in their paper, cannot cross the blood–brain-barrier (BBB), thus they used a liposome carrier to deliver the molecules to the brain tissue. After i.v. injection of 9-azido sialic acid-loaded-liposomes, the molecule could reach the brain and participate in brain metabolic glycoengineering. As a result, the newly synthesized sialic acid in the brain tissue could be modified with azide groups, which could be further labeled after i.v. injection of DBCO-Cy5.5 by SPAAC *in vivo*. Even though fluorescence imaging has many advantages, including easy handling and high resolution, its application in medical imaging is restricted due to its short penetration depth.^91^ Therefore, other imaging modalities including PET/SPECT, MRI, and ultrasound are, preferred in clinical settings, researchers have also tried to apply click chemistry *in vivo* with these imaging techniques. In 2010, Rossin *et al.* applied click chemistry *in vivo* to SPECT imaging for tumor imaging in mice.^52^

They modified anti-TAG72 monoclonal antibody CC49 with TCO, and synthesized a Tz-conjugated chelator containing ^111^In as a counterpart for SPECT imaging. They first injected a TCO–antibody to a colon cancer-bearing mouse *via* i.v. administration, and also injected Tz-^111^In similarly the next day. They obtained 4.2% of ^111^In accumulated in tumor tissues with a very high tumor-to muscle-ratio (13.1 : 1). Such two step strategies have been called ‘pretargeting’, which uses modified antibodies and their counterparts with imaging molecules or drugs.^28^

Pretargeting has certain advantages, including that more than one click molecule can be modified to one antibody, which increases the number of potential binding chances between one receptor and introduced imaging probes. Furthermore, in the case of imaging, pretargeting can provide sufficient time for circulation and accumulation of large antibodies while small imaging probes can already distribute rapidly.^92^ It means that unbound imaging molecules can be cleared faster than when they are linked to large antibodies. This can result in shortened waiting times between injection of probes and real imaging. Rossin *et al.* have obtained fine SPECT imaging of tumors using a fast Diels–Alder reaction *in vivo*, and this represents a good example of pretargeting *in vivo*.^52^

**Fig. 7:**
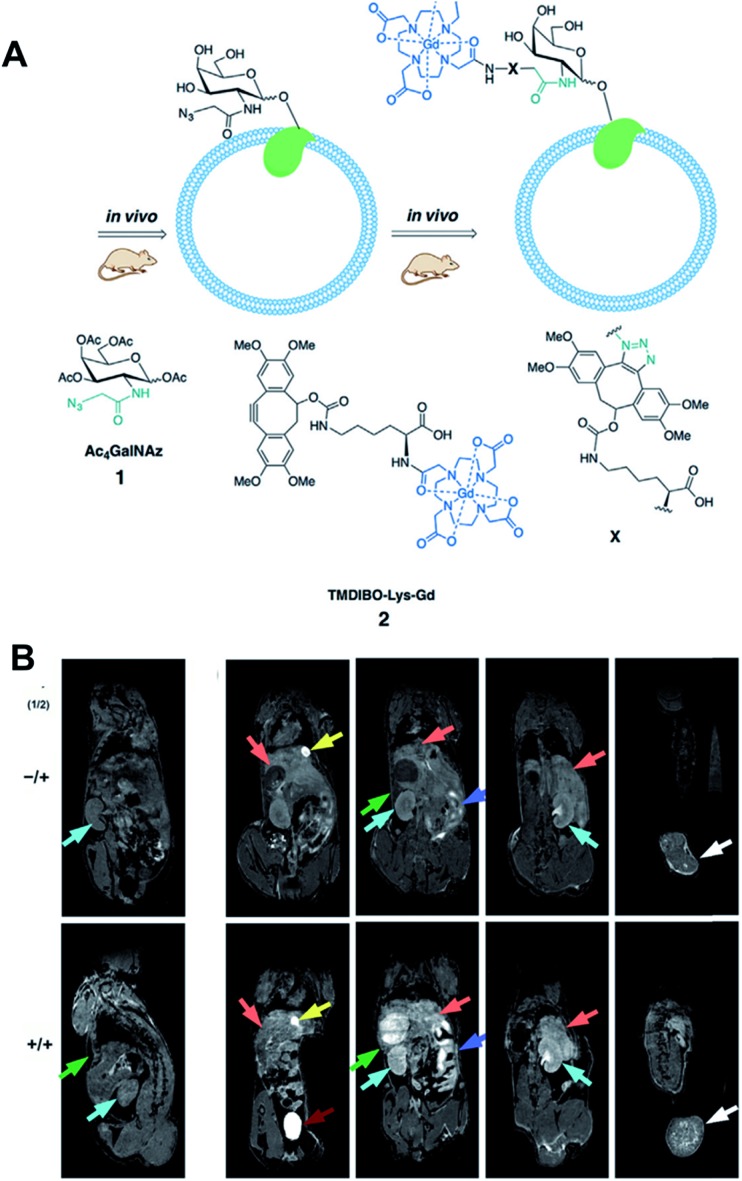
*In vivo* MR imaging of LL2 tumor glycans by copper-free click chemistry. (A) Two step strategy including i.p. injection of Ac_4_GalNAz and i.v. injection of TMBIDO-modified gadolinium into the mice. Azido-labeled glycoproteins were labeled with TMBIDO-modified gadolinium by SPAAC for MR imaging. (B) *In vivo* T1-MR images of LL2 tumor glycans labeled by SPAAC. Mice were injected with solvent vehicles (upper) or Ac_4_GalNAz (lower), and TMBIDO-modified gadolinium was injected into both mice. Images were displayed from the ventral towards the dorsal side. Tumor (white arrows), kidney (cyan), liver (orange), gut (purple), and spleen (green). Reproduced from ref. 53 with permission from John Wiley and Sons, copyright 2016.

Recently, Neves *et al.* have applied click chemistry *in vivo* to MR imaging (Fig. 7).^53^ They introduced Ac_4_GalNAz to LL2 tumor-bearing mice by intraperitoneal injection, which labeled glycans in the mouse tissues with azide groups by metabolic glycoengineering. These azide groups could be further labeled with i.v. injected TMDIBO-Lys-Gd (tetramethoxydibenzocyclooctyne-lysine-gadolinium) by SPAAC *in vivo*. Because gadolinium can change T1 relaxation rates and generate an MR signal, increased accumulation of TMDIBO-Lys-Gd could be observed by MR. Significant changes in T1 relaxivity were observed in most tissues including tumor tissues in the case of Ac_4_GalNAz-treated mice showing vigorous glycosylation *in vivo*. These results showed that the synthesized gadolinium-labeled click probe could be used for glycosylation imaging *in vivo* by MR. MR imaging has superior spatial resolution and is widely used in clinical settings, these kinds of approaches for further MR imaging with click chemistry *in vivo* have great potential.

Zlitni *et al.* have introduced a targeting method based on click chemistry *in vivo* for ultrasound imaging.^54^ Similar to the study by Rossin *et al.*, they used TCO-modified antivascular endothelial growth factor receptor 2 antibody (TCO-antiVEGFR2). VEGFR2 is overexpressed in tumor cells, thus TCO-antiVEGFR2 can bind SKOV-3 human adenocarcinoma tumor tissues in mice after i.v. injection. After that, they injected Tz-modified microbubbles filled with gas for ultrasound contrast enhancement to mice *via* tail veins. These bubbles could bind TCO-antiVEGFR2 to tumor tissues by click chemistry *in vivo*, generating ultrasound signals in tumor tissues. Quantitative data showed that these pretargeting methods provided approximately 4-fold increased signals in tumor tissues compared to non-targeted microbubbles. It showed an approximately 40% increase in value compared to that of the control microbubbles directly modified with antibodies. This type of approach using bioorthogonally modified microbubbles and pretargeting is useful to increase targeting efficacy. Using a similar strategy, Wang *et al.* performed successful ultrasound imaging of acute thrombus in rats.^55^ The results of the studies discussed are promising and demonstrate that click chemistry has great potential in biomedical imaging *in vivo*. However, strategies and the click molecules used need to be selected carefully because required doses of probes and reaction times will vary according to the types of imaging modalities and disease models.

### 3.2 *In vivo* drug delivery

Click chemistry *in vivo* is also attractive to researchers for therapy. In therapeutic applications, click chemistry was first used for synthesis or modification of drug carriers. The fast second order reaction rate constant, simplicity, and orthogonality of click chemistry are useful for polymer synthesis or site-specific modification of biological ligands during development of drug carriers.^6^ In 2012, Colombo *et al.* used SPAAC to conjugate ScFv antibodies to nanoparticles specifically. The Boons group used similar reactions during development of their antibody–drug conjugate to minimize the decrease of antibody binding affinity after modification.^22^,^93^ However, *in vivo* applications began to be used later because there was not a sufficient amount of data to be confident in the potential of click chemistry *in vivo*. Particularly, it is more delayed in drug delivery and therapy compared to imaging, because the required amount of molecules to be delivered for therapy is generally larger than that for imaging. Therefore, research using click chemistry *in vivo* for drug delivery and therapy is relatively unexplored, and more information is needed.

In 2012, Koo *et al.* demonstrated that movement and distribution of nanoparticles could be changed by click chemistry *in vivo*.^25^ They directly injected the metabolic precursor Ac_4_ManNAz into tumor tissues of mice. After the generation of azide groups on tumor cells by metabolic glycoengineering, liposomes modified with DBCO groups were injected into mice intravenously. They showed that the accumulation of DBCO-modified liposomes was significantly increased by SPAAC *in vivo* between azide and DBCO. It is evident that the azide-DBCO reaction is slower than the Tz–TCO reaction. Some researchers have pointed out that it is too slow for pretargeting *in vivo*. However, in their paper, the amount of azide groups on tumor tissues might be larger than that of specific receptors for biological ligands, which is helpful for reactions *in vivo*. In addition, nanoparticles have longer circulation times than small molecules after i.v. injection. Thus, they could provide sufficient contact time among click molecules.

**Fig. 8:**
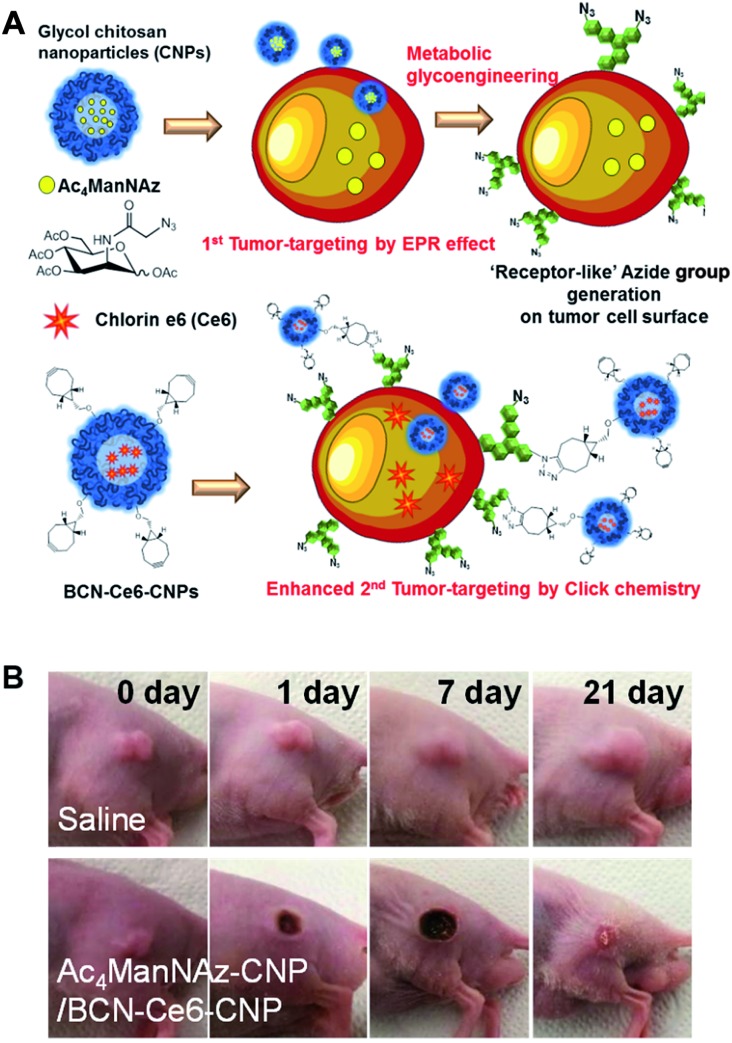
*In vivo* tumor-targeted delivery of photosensitizers and photodynamic therapy by copper-free click chemistry. (A) Azide group labelling of tumor cells by Ac_4_ManNAz-loaded nanoparticles and second tumor targeting by DBCO-modified nanoparticles containing photosensitizers. Both nanoparticles were injected into mice sequentially, and tumor-targeting was enhanced by SPAAC between azide groups and DBCO. (B) *In vivo* results of photodynamic therapy after copper-free click chemistry-based tumor-targeting. Ac_4_ManNAz-loaded nanoparticles and DBCO-modified nanoparticles containing photosensitizers were injected sequentially, and laser was irradiated over the tumor site. Reproduced from ref. 27 with permission from the American Chemical Society, copyright 2014.

Their work provides evidence, for the first time, that click chemistry *in vivo* could be used for nanoparticle delivery. This opened new avenues for these kinds of approaches for further research. However, the main disadvantage of this project was that the metabolic precursor was directly injected into the tumor tissues for pretreatment. In real clinical situations, drug delivery is frequently used to kill tumor cells of unknown location and often when intratumoral injection is impossible.^94^ To avoid the need for intratumoral injection, Lee *et al.* (the same research group) have developed an alternative strategy with i.v. injections (Fig. 8).^27^ The metabolic precursor Ac_4_ManNAz was encapsulated into glycol chitosan nanoparticles and injected intravenously into the mice. Subsequently, it was delivered to the tumor tissue by the EPR (enhanced permeability and retention) effect based on the passage of nanoparticles through fenestrated tumor vessels. The high accumulation of Ac_4_ManNAz could induce the generation of azide groups on the tumor tissues by metabolic glycoengineering. Furthermore, second nanoparticles containing drugs and BCN were again injected intravenously. They were delivered to the tumor tissue efficiently by SPAAC *in vivo* compared to bare nanoparticles or without first injection of Ac_4_ManNAz-loaded nanoparticles. Enhanced drug delivery by this two-step strategy based on metabolic glycoengineering and click chemistry *in vivo* resulted in effective tumor therapy in mice.

**Fig. 9:**
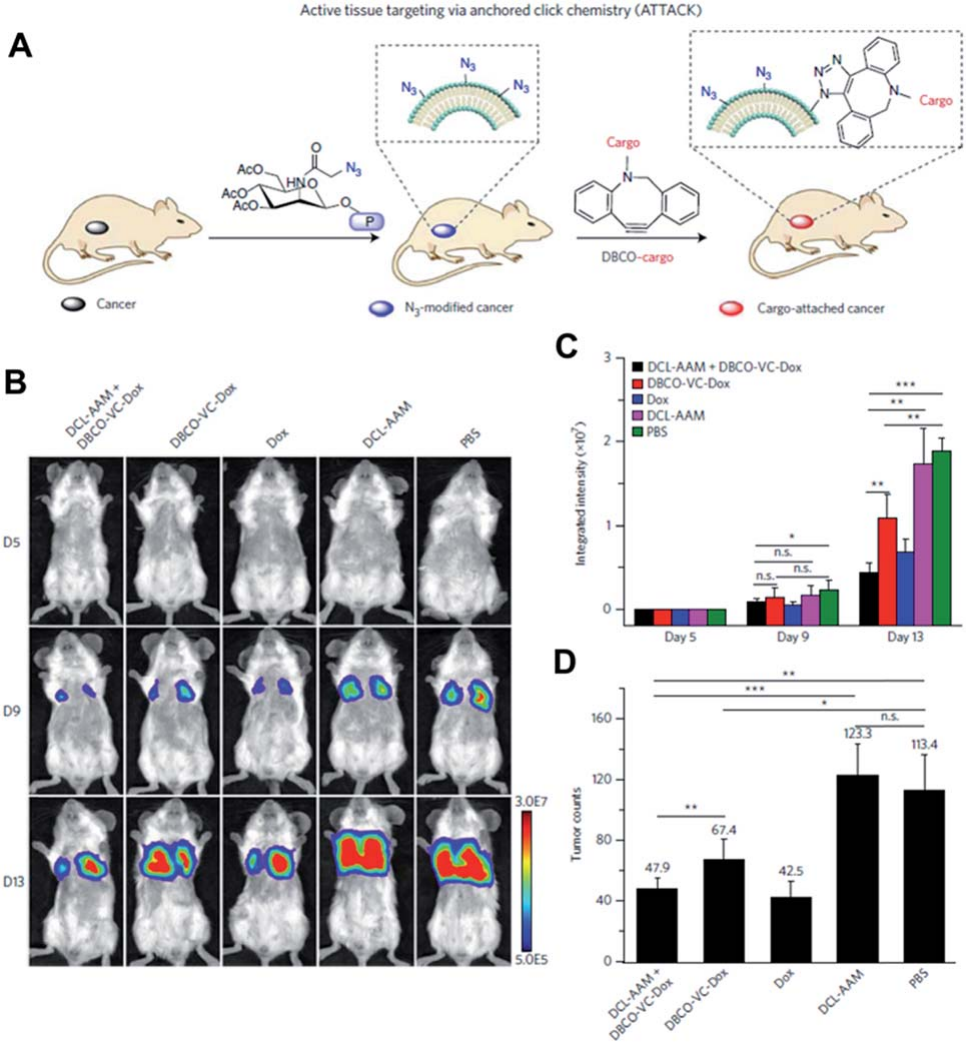
*In vivo* tumor-targeted delivery of doxorubicin and chemotherapy by copper-free click chemistry. (A) Tumor tissue labeling by a cathepsin-responsive precursor and targeted delivery of DBCO-modified cargo by SPAAC. The cathepsin-responsive precursor generated azide groups on tumor cells specifically, and they were targeted by DBCO-modified cargo. (B) *In vivo* luminescence images showing enhanced targeting of doxorubicin by SPAAC and improved therapeutic efficacy. First, the cathepsin-responsive precursor was injected into luciferase-engineered 4T1 tumor-bearing BALB/c mice. Then, DBCO-peptide-doxorubicin was injected into the mice and tumor growth was monitored by luminescence imaging. (C) Quantitative analysis of luminescence signals in (B). (D) Tumor counts in mice after click chemistry-based chemotherapy. All the numerical data are presented as mean ± s.e.m. and analyzed by one-way ANOVA (Fisher; 0.01 < *P ≤ 0.05; **P ≤ 0.01; ***P ≤ 0.001). Reproduced from ref. 56 with permission from Springer Nature, copyright 2017.

The Cheng group is also interested in using click chemistry *in vivo* for drug delivery.^56^ They achieved selective metabolic glycoengineering of cancer cells by modifying Ac_4_ManNAz with *N*,*N*′-l-diacetyllysine that could be cleaved by histone deacetylase and cathepsin L (Fig. 9). These enzymes are overexpressed in cancer cells, so that the modified precursor can be selectively activated after cellular uptake, introducing azide groups on the surface of cancer cells by metabolic glycoengineering. They showed that large amounts of azide groups were introduced by i.v. injection of the modified precursor into nude mice bearing subcutaneous LS174T tumors (primary colon tumors). In contrast with the study by Lee *et al.*, using nanoparticles, they directly modified doxorubicin, a representative anticancer drug. The amine groups of doxorubicin were modified with DBCO and dipeptide (Val and Cit) using a self-immolative *p*-aminobenzylcarbamate crosslinker. After i.v. injection of the modified doxorubicin, DBCO groups can be conjugated with azide groups on the surface of cancer cells by SPAAC *in vivo* resulting in increased accumulation in the tumor tissues.

After cellular uptake, free form doxorubicin was generated to kill cancer cells through cleavage of the dipeptide by cathepsin B. The apoptosis index in the tumor tissue was found to be 33.5% using this strategy. It was much higher than the 18.3% found in the case without metabolic glycoengineering, thus improving tumor targeting by SPAAC *in vivo*. The group also showed the improved anticancer efficacy of this strategy in MDA-MB-231 triple negative breast tumor and 4T1 lung metastases models. Particularly, doxorubicin was modified and selectively activated in tumor tissues resulting in lowered toxicity to bone marrow and spleen. These overall studies contain new challenges and promising data demonstrating that click chemistry *in vivo* is useful for drug delivery. Better applications are expected in the future.

In 2019, Feng *et al.* introduced an assembly of upconversion nano-photosensitizers based on click chemistry *in vivo*.^57^ This system is composed of upconversion NPs modified with Tz groups (UCNP-Tz) and a norborane (NB)-conjugated photosensitizer, rose bengal (RB). The UCNP-Tz was further modified with folate (FA) to target the receptors on tumor cells. They injected the UCNP-Tz-FA into MCF-7 tumor-bearing mice, and the particles accumulated in tumor tissue by the EPR effect and binding to FA receptors. After that, RB-NB was injected into tumor tissue directly. Because the excitation wavelength of RB is 530–560 nm, it is not reactive upon laser irradiation with wavelength in the NIR region.^95^ By iEDDA between Tz and NB in tumor tissue, RB-NB can be conjugated to the surface of UCNP-Tz-FA, and it can be stimulated by energy transfer from UCNP irradiated by laser with 980 nm. The *in vitro* and *in vivo* data showed click chemistry-triggered energy transfer and the following regeneration of the photodynamic effect for therapy. The authors insisted that this ‘off’–‘on’ system can minimize the unintended phototoxicity during photodynamic therapy. This study is rationally designed and provides new potential of the click chemistry *in vivo*, but intratumoral injection of RB-NB may limit wide application similar to the study of Koo *et al.* as mentioned above.^25^

A recent study of Li *et al.* showed application of click chemistry *in vivo* to cardiac cell therapy.^58^ To cure myocardiac infarction (MI) by cell therapy, large numbers of stem cells need to arrive at the injury site in the heart. For this purpose, the authors used two kinds of antibodies, CD41 and CD34 which bind platelets and endogenous stem cells, respectively. They modified CD41 antibody with PEG and DBCO, and injected it into MI mice intravenously. Then, the DBCO-PEG-CD41 binds platelets in blood, which accumulate on the MI region in the heart by their homing ability. After that, granulocyte colony-stimulating factor was administered to the mice to stimulate and release endothelial progenitor cells (EPCs). Next, azide and PEG modified CD34 (Az-PEG-CD34) was also injected intravenously. It binds DBCO-PEG-CD41 in MI tissue area by SPACC *in vivo*, and recruits EPCs to the region for therapy. Injection of DBCO-PEG-CD41 and Az-PEG-CD34 increases the amount of DiR-labeled EPCs accumulated in the heart more than five-fold. This click chemistry-based pre-targeting strategy attenuated fibrosis and increased CD34+ cells in the MI region showing improved therapy *in vivo*.

## 4. Click chemistry *ex vivo*

### 4.1 *Ex vivo* diagnosis

Recent studies have highlighted the potential of click chemistry *ex vivo* in providing valuable information for understanding tissue development, monitoring disease state, and diagnosing diseases. For instance, Rong *et al.* performed glycoproteomic analysis of intact and hypertrophic hearts using click chemistry with Ac_4_ManNAz for metabolic labelling of glycomes in hearts.^59^ They injected Ac_4_ManNAz by intraperitoneal injection into Sprague-Dawley rats. Later sialylated glycans were labeled with alkyne-biotin using the CuAAC reaction assisted by the ligand BTTAA. With this approach, they confirmed successful metabolic incorporation of an azide tag to the glycome of the mouse model by western blotting of lysates and fluorescence imaging of isolated cells using alkyne-Fluor 488. By using a Langendorff-perfusion system, they successfully performed fluorescence imaging of intact hearts with a SPAAC copper-free click reaction with aza-dibenzocyclooctyne-Fluor 488 (DBCO-F488). They found an intense fluorescence signal on the cell surface which was more pronounced at the cell–cell surface and on the transverse tubule (T-tubule) of the heart tissue. Furthermore, they applied this method to study cardiac glycome changes depending on pathogenesis. With SPAAC reactions, they found a significant increase of fluorescence signal in the heart of the ISO-treated cardiac hypertrophy mouse than in the heart of the saline-treated control mouse. In addition, through gel-based proteomic study assisted by the click reaction with alkyne-biotin and tandem mass spectroscopy, they found 21 and 18 proteins uniquely identified in the hypertrophic mouse and the healthy control mouse, respectively. In addition, they found that NCAM1 and α_2_M, known to be proteins relevant to hypertrophic disease, were mostly up-regulated among sialylated proteins.

**Fig. 10:**
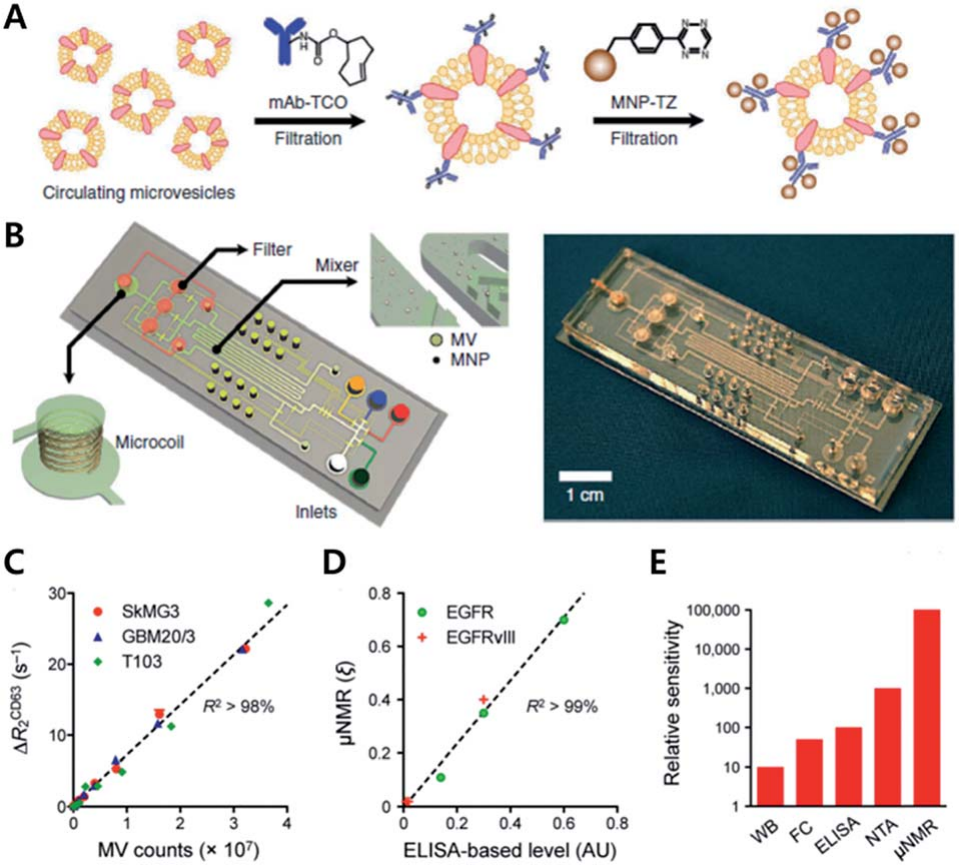
Protein profiling on microvesicles *ex vivo* by copper-free click chemistry. (A) Schematic illustration of labelling of extravesicular markers with copper-free click chemistry to maximize magnetic nanoparticle binding to target markers for μNMR measurement. (B) Microfluidic system for on-chip analysis of markers on microvesicles (MVs). (C) Sensitive detection of MV numbers from three different cell lines with μNMR assay. (D) Comparable sensitivity between μNMR measurement and fluorescence ELISA for expression level analysis of EGFR and EGFRvIII markers on MVs. (E) Comparison of the detection sensitivity of MVs in the sample (WB: western blotting, FC: flow cytometry, NTA: nanoparticle tracking analysis). Reproduced from ref. 60 with permission from Springer Nature, copyright 2012.

Since many cancers release membrane-bound small microvesicles (MVs) into peripheral circulation, an analytical tool of MVs holds as a promising diagnosis method for diseases such as glioblastomas (GBMs). In 2012, the Lee group reported on a microfluidic system combined with iEDDA type click chemistry and miniaturized micro-nuclear magnetic resonance (μNMR) for analyzing MVs from the blood of a GBM patient (Fig. 10).^60^ In their study, they first targeted protein markers of MVs with TCO modified antibodies. Later, the antibodies were coupled with Tz modified magnetic nanoparticles (MNPs) to increase the magnetic signal. With this two-step bioorthogonal approach, they successfully increased the signal >300% compared to that of direct MNP–antibody conjugation. They confirmed that the system had a low error rate (<1% instrumental error) showing excellent agreement with the fluorescence ELISA method. In addition, they confirmed that the system has great detection sensitivity (from 10^4^- to 10^2^-fold), higher than other analytical methods such as western blotting, ELISA and NTA (nanoparticle tracking analysis) because bioorthogonal click chemistry allowed dense packing of MNPs on MVs.

Analysis of protein markers such as HSP90 (MV maker), CD41, and MHCII (host cell marker) positive MVs revealed that MVs indeed reflected the protein profiles of parent cells. Furthermore, they could monitor the efficacy of drug treatments such as temozolomide and geldanamycin for GBM disease. They confirmed that TMZ treatment did not induce significant changes in protein markers such as CD63, EGFR, or EGFRvIII. On the other hand, geldanamycin induced a significant decrease in EGFR and EGFRvIII expression in both cells and MVs.

**Fig. 11:**
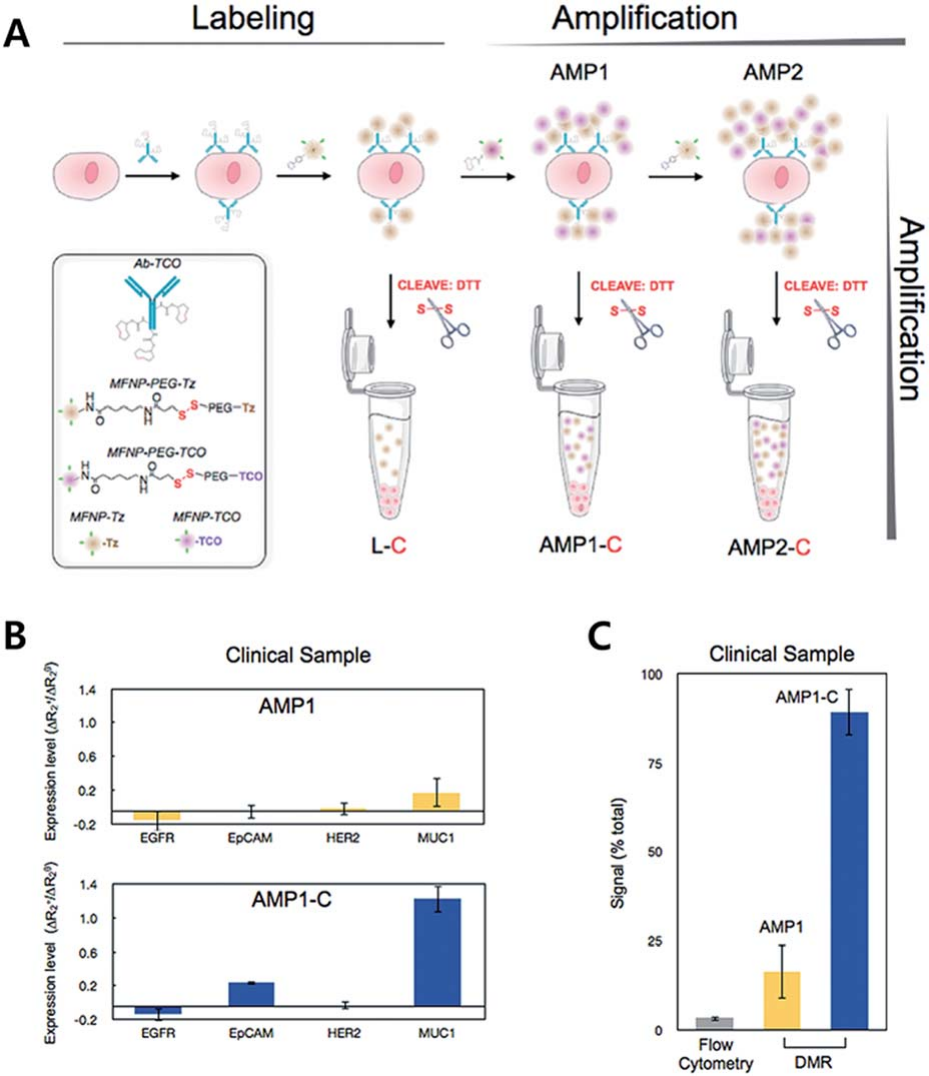
iEDDA type signal amplification strategy for bioorthogonal diagnostic sensing *ex vivo*. (A) Schematic illustration of bioorthogonal signal amplification strategy for biomarker analysis with copper-free click chemistry. (B) Expression level profiling of four different markers (EGFR, EpCAM, HER2, and MUC1) at human clinical ascites from pancreatic cancer. (C) The DMR system offers better sensitivity than the flow cytometry method for MUC1 detection in clinical samples. Reproduced from ref. 61 with permission from American Chemical Society, copyright 2012.

For diagnostic purposes, the limit of detection (LOD) for the technology is crucial. There have been various attempts to amplify signals to lower the LOD. The Weissleder group has reported an iEDDA type signal amplification strategy for nanoparticle-based bioorthogonal diagnostic sensing assisted by a miniaturized diagnostic nuclear magnetic resonance device (DMR) (Fig. 11).^61^ Briefly, antibodies were labeled with TCO, and then they were coupled with Tz modified magneto-fluorescent nanoparticles (MFNP-Tz). The generated signal could be amplified again with TCO modified MFNP (MFNP-TCO) to form multiple MFNP layers. Since the linker between MFNP and Tz or TCO is cleavable, MFNPs are released from each other. Transverse relaxation rate (*R*_2_) signals can then be measured by the DMR system. Firstly, they tested the amplification method on SK-OV-3, human ovarian carcinoma overexpressing HER2, with anti-HER2 antibodies (trastuzumab). They confirmed that the PEG linker between MFNP and TCO or Tz resulted in a higher signal to noise ratio. More importantly, alternative labelling with the MFNP-TZ/MFNP-TCO amplification method resulted in a significantly higher signal than single antibody labelling with MFNP-TZ. Validation of the method using clinical samples from pancreatic cancer patients with EGFR (epidermal growth factor receptor), EpCAM (epithelial cell adhesion molecule), HER2, and MUC1 (mucin-1) antibodies, indicated that the method could be used for profiling scarce cells from clinical samples.

### 4.2 *Ex vivo* mechanism study

Considering the importance of protein synthesis for understanding the plasticity of synaptic connections, Dieterich *et al.* metabolically introduced noncanonical amino acids such as azidohomoalanine (AHA) and alkyne-bearing amino acid homo-propargylglycine (HPG) into newly synthesized proteins as methionine surrogates. These proteins were then chemoselectively labeled by click chemistry with Texase Red-PEG-alkyne (TRA) and fluorescein-PEG-azide (FKA) to study protein dynamics in neuronal systems.^62^ They confirmed that newly synthesized dendritic neuronal proteins could be labeled using click reactions with AHA/TRA or HPG/FKA pairs with copper catalysts and triazole ligands, both in dissociated neurons and in a slice of brain tissue. By pulse charging HPG followed by AHA and labelling with corresponding TRA and FKA, they labeled newly synthesized proteins at two different time points. After demonstrating the labelling method, they used quantum dots (QDs) with copper-free click reactions between AHA-tagged proteins and DIFO (a difluorinated cyclooctyne)-biotin, followed by streptavidin-QD treatment to monitor individual newly synthesized proteins. They found that newly synthesized proteins generally have higher mobility than other neuronal membrane proteins. Furthermore, AHA-tagged proteins were slower in synapses than outside synapses.

**Fig. 12:**
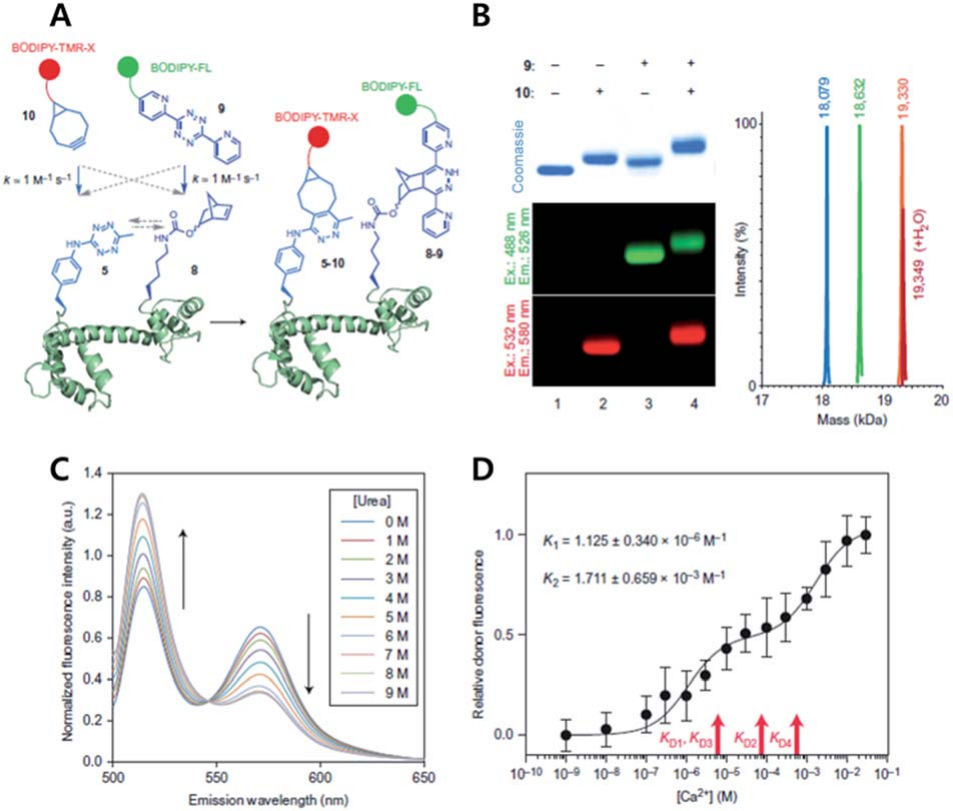
Copper-free click chemistry for protein conformational change study. (A) Schematic illustration of double-site specific labelling strategy with click chemistry. (B) Fluorescence scanning images of SDS-PAGE result of CaM labeled with BODIPY-TMR-X (10) or/and BODIPY-FL (9) *via* iEDDA reaction. (C) FRET signal changes between 10 and 9 according to concentrations of urea. (D) Relative donor fluorescence changes according to concentrations of Ca^2+^. Reproduced from ref. 63 with permission from Springer Nature, copyright 2014.

Wang *et al.* have reported a method for incorporating multiple distinct noncanonical amino acids with proteins by evolving a pyrrolysyl–tRNA synthetase (PylRS/tRNA) pair with orthogonal ribosome (ribo-Q1) (Fig. 12).^63^ They used saturation mutagenesis libraries consisting of 10^8^ membered Pyl tRNA (N8)_XXXX_ with eight different nucleotides in the anticodon stem loop. Desired tRNAs were selected through negative selection (to exclude Pyl tRNA(N8)_XXXX_ as the substrate for both endogenous synthetase and decoded on ribo Q1) and positive selection (aminoacylated with an unnatural amino acid by PylRS and decoded efficiently on ribo-Q1). They confirmed that the evolved Pyl tRNA_XXXX_ had improved quadruplet decoding efficiency and specificity which allowed efficient incorporation of unnatural amino acids into the proteins.

The study also demonstrated that unique chemical handles, including azides, alkenes, alkynes, benzophenones, and tetrazines, could be incorporated into the proteins of interest with a combination of PylRS substrates and four distinct substrates for *Mj*TyrRS variants. They confirmed that their system had better efficiency than the *Mj*TyrRS/tRNA_CUA_ variant and PylRS/tRNA_UUA_ or PylRS/tRNA_UCA_. With the system, they incorporated two different noncanonical amino acids containing Tz and norbornene, into calmodulin (CaM) proteins by combination of *Mj*TetPheRS/tRNA_CUA_ and a NorKRS/evolved Pyl tRNA_UACU_ pair along with the corresponding noncanonical amino acid. They confirmed that there was no intramolecular reaction between iEDDA reaction pairs on the proteins. They successfully labeled corresponding noncanonical amino acids with BODIPY-FL tetrazine or BODIPY-TMR-X bicyclononyne with a rate constant of around 1.0 M^–1^ s^–1^. In addition, they labeled CaM protein sites specifically with FRET fluorophore pairs using copper-free click reactions and successfully studied conformational change of the proteins within a domain resulting from sequential ligand binding events by monitoring FRET signal changes.

### 4.3 Nucleotide ligation and CRISPR Cas9 system

Nucleotides such as DNA or RNA are generally linked by ligase, but their chemical ligation is also an interesting example of click chemistry. The Brown group have pioneered in this field and developed diverse nucleotide structures by click chemistry until now.^96^,^97^ In a recent study, this group demonstrated the utility of click chemistry-mediated nucleotide ligation in the CRISPR Cas9 system.^64^ Genome editing by this system needs a single-guide (sg) RNA which enables Cas9 protein to cleave a specific DNA sequence. However, the high cost and long time involved in synthesizing sgRNA have limited wider application of CRISPR Cas9. Taemaitree *et al.* designed a new type of sgRNA composed of a variable 20-mer DNA-targeting region and fixed the 79-mer Cas9-binding region. These two regions were synthesized separately, and the ends of 20-mer and 79-mer RNAs were modified with propargyl (alkyne) and azide groups, respectively. They were conjugated with each other by CuAAC ligation for 1–2 hour. Then, the on-target activity of the resulting clicked sgRNA was evaluated in live U2OS cells. The clicked sgRNA successfully mediated indel formation similar to control sgRNA prepared by *in vitro* transfection as shown in the T7E1 assay showing that the activity was not reduced after CuAAC ligation. In addition, to prevent the potential risk of the copper catalyst, they also performed ligation using DBCO groups instead of alkynes, and showed that SPAAC between azide and DBCO groups could achieve similar results. This study demonstrates that click chemistry can be a useful tool to improve other promising technologies such as CRISPR Cas9 in biology and biomedical science.

To overcome the limitations of the broadly applied Cu(i)-catalyzed azide–alkyne cycloaddition for the labeling of nucleic acids, multiple different studies for applying copper-free cycloaddition reactions to label nucleic acids have been reported. For example, the Kath-Schorr group reported site specific labeling of RNA oligonucleotides *via* the iEDDA reaction between norbornene and tetrazine-fluorophore conjugates.^45^ In this study they synthesized a clickable RNA nucleotide *via* norbornene-modified uridine phosphoramidite and performed a successful iEDDA reaction with multiple different tetrazine-fluorophores (Oregon Green 488, and ATTO647) both *in vitro* and in cells. In 2016, they reported a cyclopropene-modified ribonucleotide TPT3CP TP for site specific modification of RNA nucleic acids *via in vitro* transcription using the (d)TPT3–dNaM system.^46^ They confirmed the iEDDA reaction of cyclopropene-modified oligonucleotides with the tetrazine-fluorophore conjugate *via* LC-MS, HPLC and PAGE analysis. Very recently, the Wagenknecht group reported copper-free dual labeling of the DNA molecule *via* incorporation of two different bioorthogonally reactive groups, 1-methylcyclopropenes and 1,2,4-triazines. They synthesized a 1,2,4-triazine containing nucleoside *via* modification on the 5-position of 2′-deoxyuridine triphosphate and a 1-methylcyclopropene containing nucleoside *via* modification on the 7-position of 7-deaza-2′-deoxyadenosine triphosphate. They demonstrated successful incorporation of the bioorthogonal nucleoside *via* primer extension and the resulting oligonucleotide product was labeled with two different fluorescent dyes such as BCN-rhodamine and tetrazine-BODIPY.^47^

## 5. Conclusions

In this review, we have introduced important researches based on copper-free click chemistry *in vitro*, *in vivo* and *ex vivo* for biomedical applications. Click chemistry enables chemical conjugation between two molecules with specificity and fast second order reaction rate constant under aqueous conditions without interference from other surrounding molecules. It means that artificial chemical reactions are now possible on cell surfaces, in cell cytosol, and within the body. Many studies and papers have demonstrated the great potential of click chemistry not only in chemical synthesis but also for biological and biomedical applications. However, to obtain expected results in studies, we need to have an in-depth understanding of the inherent characteristics of click chemistry so as to select the optimal chemical groups for the intended purpose of the research. Particularly, for applications *in vivo*, there is not sufficient time for conjugation in many situations, including i.v. injections. For these cases, the importance of reaction time should be considered in more detail.

In 2012, the Weissleder group pointed out that favorable pharmacokinetics and increased circulation time of injected molecules by conjugation with polymers of high molecular weight enhanced the reaction of click chemistry *in vivo*.^98^ After i.v. injection into mice, Tz groups showed a greater than 10-fold increase in the efficiency of click chemistry *in vivo* when they were attached to a 10 kDa dextran polymer compared to that of direct attachment to a fluorescent dye. Koo *et al.* have also shown the effects of pharmacokinetics of injected molecules on click chemistry *in vivo*.^99^ They conjugated Tz groups with fluorescent dyes of various chemical structures. In mice, the change of charge or hydrophobicity changed their biodistribution, circulation, and secretion. When they compared the efficiency of click chemistry *in vivo* using these molecules, they found that the efficiency was highly dependent upon the kinetics *in vivo* even though all of them contained the same Tz groups. These results suggest that we need to consider all molecules and situations carefully to use click chemistry successfully.

Besides the second order reaction rate constant, the stability of click molecules under special conditions is also important to consider. Generally, chemical groups with superior reactivity and fast second order reaction rate constants show relatively low stability under many conditions, which is reasonable from a chemical perspective. In 2017, the Prescher group focused on Staudinger ligation and introduced cyclopropenones with high stability and improved reactivity with phosphines.^100^ They were stable after site specific genetic incorporation and enabled sequential labeling of proteins by a bioorthogonal reaction. In 2011, Karver *et al.* synthesized 12 kinds of Tz derivatives and tested their stability in serum.^101^ After 10 hours of incubation in FBS, two of them retained their reactivity almost perfectly while one molecule completely lost its reactivity. Murrey *et al.* also showed that azide groups were completely stable inside cells for more than one day. However, only 6% of BCN groups remained active under similar conditions, thus demonstrating different stabilities of click molecules.^102^

Despite these limitations, the number of applications for click chemistry in many fields continues to grow every year.^103^ This trend is supported by the development of better candidates for click chemistry. For example, to further increase second order reaction rate constants, Darko *et al.* recently developed a dioxolane-fused TCO (d-TCO) group which provides a second order rate constant *k*_2_ of 366 000 M^–1^ s^–1^ with 3,6-dipyridyl-*s*-tetrazine.^10^ In addition, the Devaraj group used a cyclopropene group that could participate in iEDDA reactions with Tz.^76^ It may be an alternative molecule of TCO with a similar second order reaction rate constant but smaller size for specific purposes. Based on these findings, we consider click chemistry to be emerging as a valuable technique in biomedical fields as well as organic chemistry. The scope of its application is expected to be even broader in the future with further advances in chemistry.

## Conflicts of interest

There are no conflicts to declare.
